# Neglected, Perineural, Chronic, Extra-articular, Lateral, Gangliomatous Synovial Knee Cyst With Peroneal Neuropathy: A Case Report

**DOI:** 10.7759/cureus.71490

**Published:** 2024-10-14

**Authors:** Lavindra Tomar, Gaurav Govil

**Affiliations:** 1 Department of Orthopedics, Max Super Specialty Hospital, Delhi, IND

**Keywords:** cyst, foot drop, intra-articular, knee, magnetic resonance imaging, meniscal cyst, perineural

## Abstract

Lateral knee cysts are relatively common synovial swelling. They are commonly benign swelling due to synovial fluid herniation from the joint. They are usually asymptomatic and in most cases are so small that only magnetic resonance imaging (MRI) can confirm the diagnosis. A synovial cyst of size more than 4 cm with nerve compression leading to peroneal neuropathy has grave clinical implications.

We report an unusual case of a large, neglected, symptomatic, chronic, lateral synovial cyst with foot drop in a 67-year-old male. He presented after almost two months of onset of weakness in the right lower limb. A cystectomy with nerve exploration and decompression was done. The functional outcome for the knee was graded to be good based on the Lysholm knee scoring scale, with no instability or restricted movements of the knee. There was no recurrence of cysts at one year. The dorsiflexion of the foot improved only partially due to the delay in treatment, though it allowed him a bipedal gait with the support of a foot drop splint.

The lateral synovial cyst with extra-articular component approached with an “early” open cystectomy and nerve decompression may preserve the knee function with good functional outcomes. Any delay in treatment may impact the overall outcomes. An early intervention has favored a better recovery outcome for both knee function and neuropathy.

## Introduction

Ganglion synovial cysts are common cystic lesions along the knee. The common causes for cyst formation include synovial herniation from joint or collection of fluid in the adjoining bursa, tendons, and ligaments. The knee may present with an extra-articular component along with an intra-articular pathology with the majority of the synovial cysts [1]. The differential diagnosis includes meniscal cyst and periosteal or intra-osseous cysts. The large cysts may present with compressive symptoms. Peroneal neuropathies are one of the most common peripheral neuropathies of the lower limb [2]. The peroneal nerve entrapment in the proximal tibia-fibular region due to a synovial cyst has been infrequently reported [3].

Perineural cysts are normally small in size and rarely present as extra-articular knee swelling. An extra-articular, large, lateral synovial cyst of size more than 4 cm has been infrequently reported. The synovial cysts are normally asymptomatic; however, foot drop, being a dramatic event, requires urgent attention [4].

In this report, we present an unusually large, lateral, neglected, symptomatic, chronic, extra-articular, perineural synovial gangliomatous cyst with peroneal neuropathy for management. An open cystectomy with decompression of the peroneal nerve was done. The functional outcome was graded as good. The delay in treatment of the neglected cyst resulted in partial recovery of paresis, although the patient maintained an ambulatory status with a foot drop splint.

## Case presentation

A 67-year-old male presented with a history of swelling over the right knee, observed for the past one and a half years. There was a complaint of mild pain along with the swelling for a duration of about one year. He took self-medications for pain from over the counter with no significant relief from the swelling. The size of the swelling remained static for almost a year with a history of progressive increased swelling in the preceding two months and has remained the same for the last two months. There was no past history of trauma, fever, or joint pains. He walked with minimal difficulty initially, though bending of the right knee was uncomfortable at the extreme of flexion. He could not recall any injury to his right knee. The ambulation required support walking on a stick for two months. No other significant medical illness was reported.

**Figure 1:**
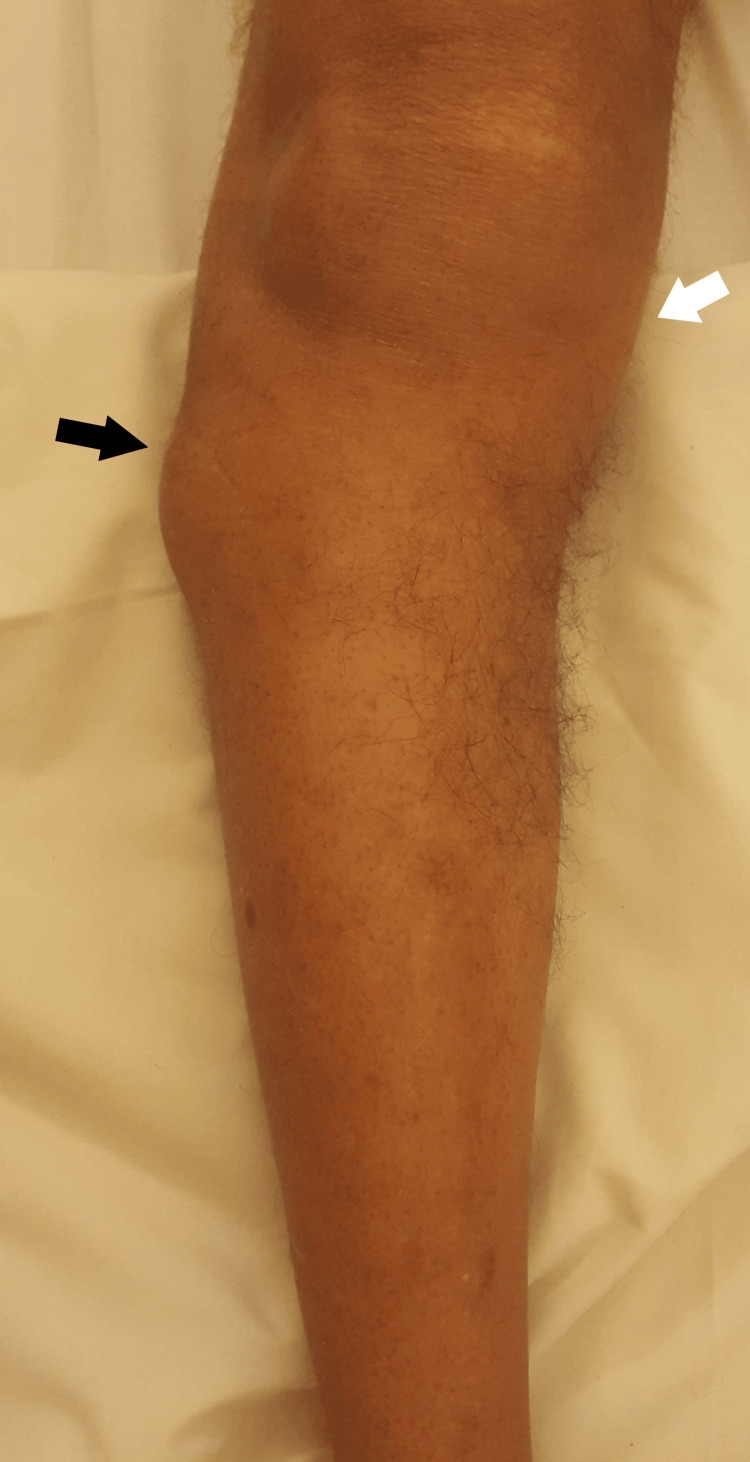
A 67-year-old male with right knee varus angulation (marked with a white arrow) and swelling on the lateral aspect of the proximal tibia (marked with a black arrow)

On local examination, the right knee had a localized single swelling along the lateral joint line and lateral plateau of the tibia, which measured approximately 4 cm x 4 cm in size. The swelling was non-tender, irreducible, non-pulsatile, firm in consistency, with normal overlying local temperature. The swelling became taut and prominent on the flexion of the knee (Figure 1).

**Figure 2:**
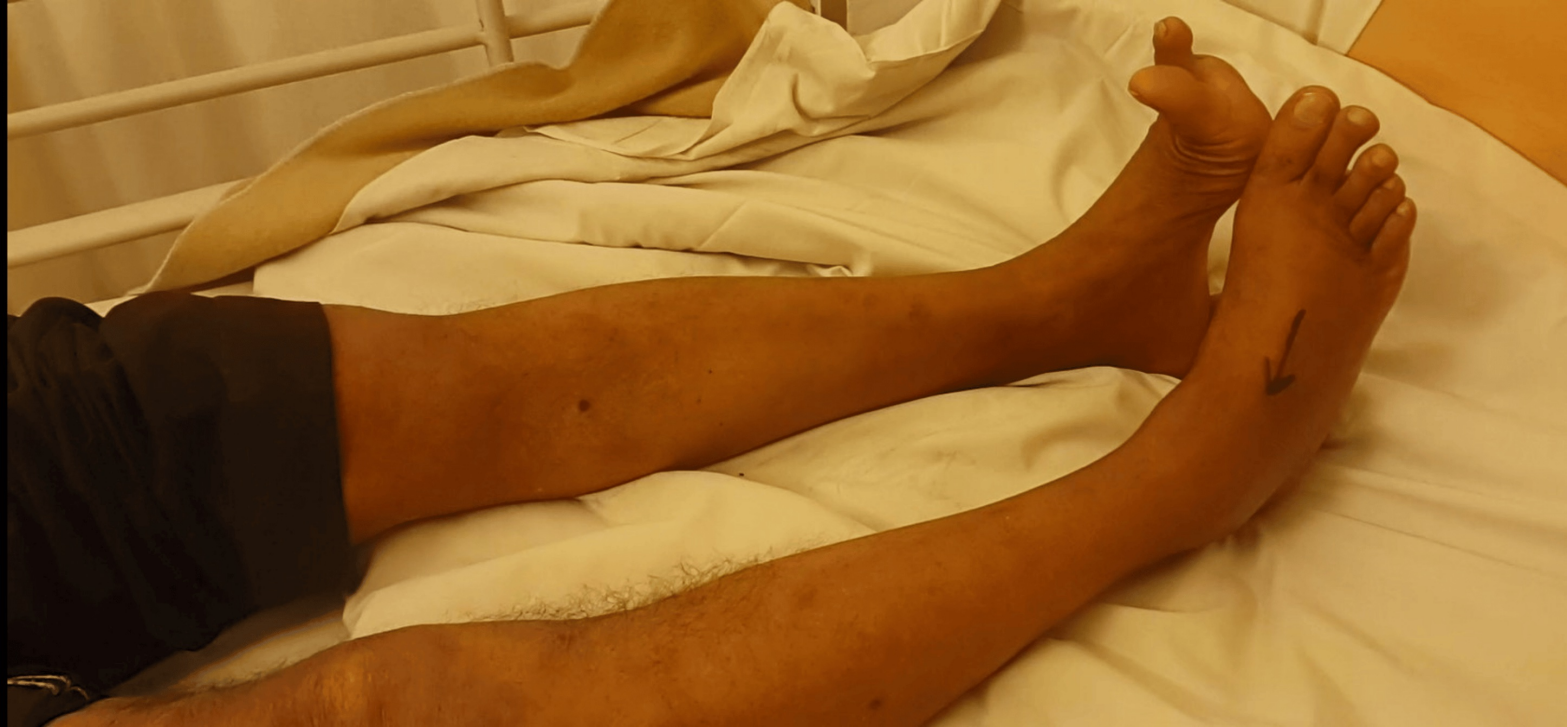
Dorsiflexion of foot on right lower limb shows weakness and foot drop

The range of motion for the right knee was terminally painful with restriction of terminal 10° flexion. There was weakness of dorsiflexion of the right foot with abnormal paresthesia in the outer aspect of the right foot (Figure 2).

**Figure 3:**
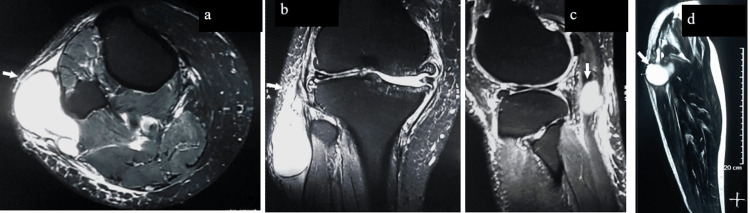
The MRI of the right knee shows an axial T1-weighted image (a), which demonstrates a uniformly hypointense mass (arrow), a hypointense lesion (b) on the coronal MR image, and a sagittal T2-weighted image (c) demonstrating a tubular bright cystic mass (arrow) just posterior to the tendon, as well as a perineural extra-articular cyst on the posterolateral aspect of the knee (d)

He walked with a high steppage gait with a stick support. Plain radiography of the right knee in anteroposterior and lateral view showed osteophytic outgrowth at medial tibial plateau with decrease in joint space with no erosive pathology and a soft tissue component along the margins of the lateral tibial plateau. The magnetic resonance imaging (MRI) of the right knee showed degenerative changes, subchondral edema in the femoral and tibial condyles, a degenerative tear of the anterior horn of the medial meniscus, and a multiloculated cystic collection along the posterolateral aspect of the knee measuring 4 cm cranio-caudally, likely a synovial gangliomatous cyst, with a normal lateral meniscus (Figure 3).

The routine hematological investigations and acute phase reactants were within normal limits. There were no symptoms of instability. A nerve conduction velocity (NCV) test for the lower limbs was done with motor NCS for bilateral common peroneal and posterior tibial nerves (Table 1).

**Table 1:** Motor NCS EDB: extensor digitorum brevis; AH: abductor hallucis

Nerve/sites	Latency, ms	Amplitude, mV	Duration, ms	Area, mVms	Distance, cm	Velocity, m/s	
Right common peroneal - EDB							
Ankle	0.00						
Fibula head							
Path knee - ankle							
Left common peroneal - EDB							
Ankle	5.00	4.5	5.30	12.3			
Fibula Head	13.20	4.4	6.40	13.7	38	46.3	
Path Knee - ankle							
Right tibial (knee) - AH							
Ankle	4.85	5.6	5.15	13.1			
Knee	14.85	3.3	7.45	11.6	41	41.0	
Path 3 - ankle							
Left tibial (knee) - AH							
Ankle	6.35	7.0	6.40	21.6			
Knee	17.60	5.6	7.70	21.3	41	36.4	
Path 3 - ankle							

The NCV test for sensory NCS for bilateral sural nerves was done (Table 2).

**Table 2:** Sensory NCS

Nerve/sites	Rec. site	Latency, ms	Pk amp, µV	Amp Pk-Pk, µV	Duration, ms	Distance, cm	Latency difference, ms	Velocity, m/s
Left sural								
Medial foot	Ankle	3.4	13.8	8.5	2.5	14	3.4	41.2
Right sural								
Medial foot	Ankle	2.9	13.0	6.5	2.3	14	2.9	48.3

It confirmed a common peroneal neuropathy with a non-recordable compound muscle action potential (CMAP) in the right common peroneal nerve, along with normal distal latencies, normal CMAP amplitudes, and normal conduction velocities in the left common peroneal and bilateral posterior tibial nerves. The right common peroneal F waves were not recordable (Table 3).

**Table 3:** F waves EDB: extensor digitorum brevis; AH: abductor hallucis

Nerve	Min F Lat, ms	Max F Lat, ms	Mean Flat, ms
Right common peroneal - EDB	0.00	0.00	0.00
Right tibial (knee) - AH	59.40	64.45	62.63
Left common peroneal - EDB	50.50	59.65	54.97
Left tibial (knee) - AH	56.65	64.75	60.73

**Figure 4:**
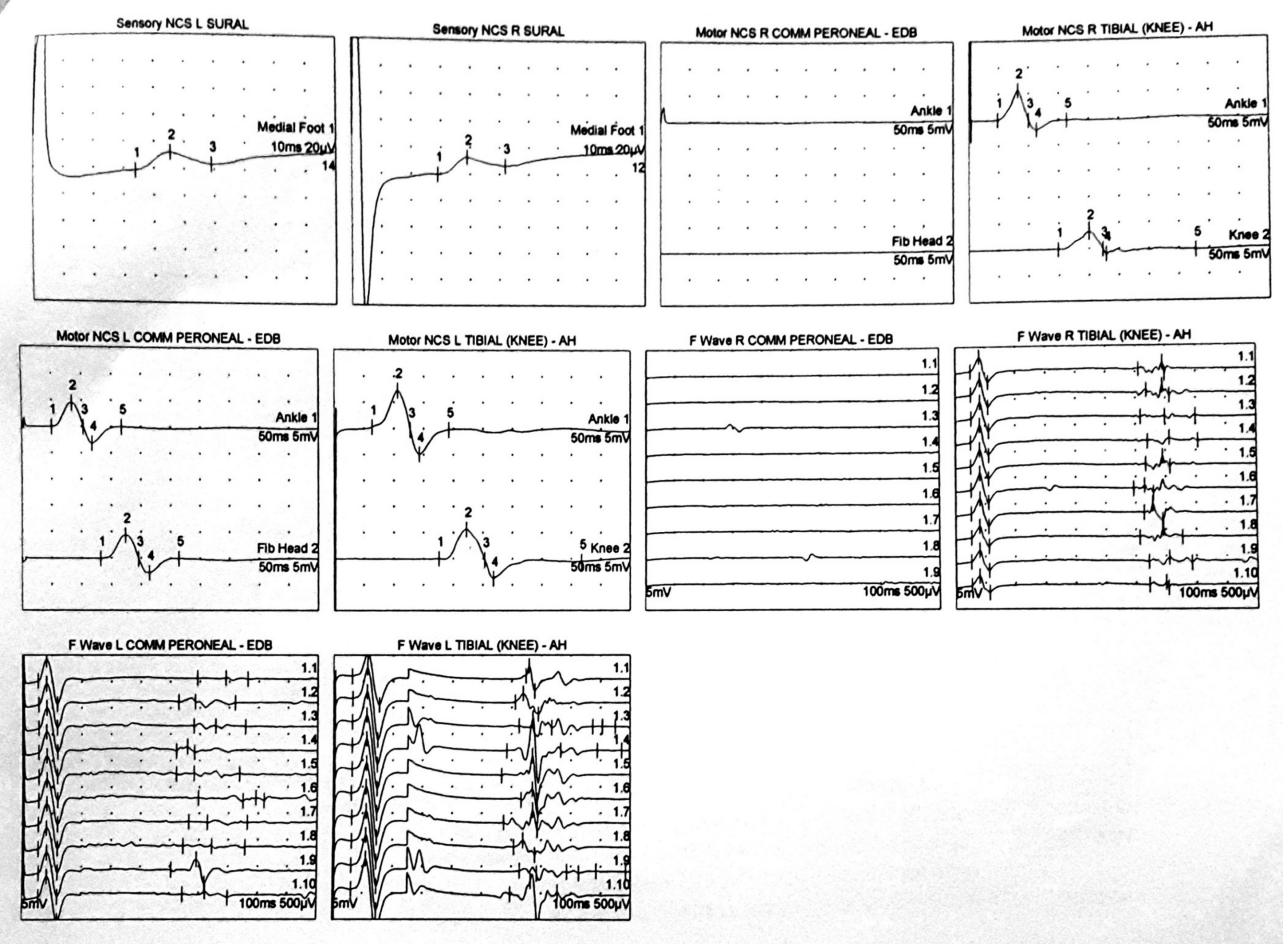
Right common peroneal F waves are non-recordable

However, F waves showed normal latency and consistency in all other tested nerves (Figure 4).

**Figure 5:**
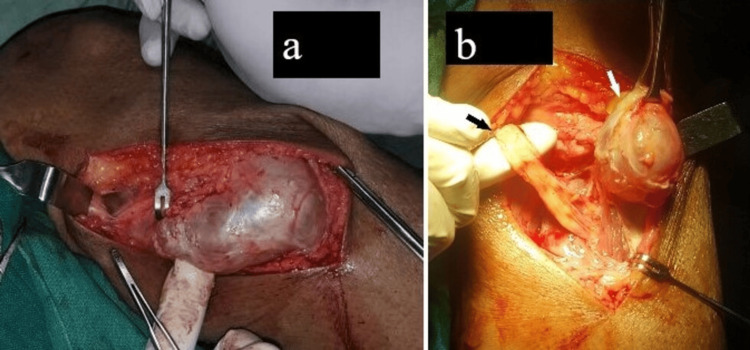
The pre-operative clinical image of the right knee shows (a) a synovial glistening, dirty white cystic swelling (b) in close proximity to the common peroneal nerve

He was advised for surgical management of the cyst with nerve decompression. The complete excision of the cyst along with nerve exploration and decompression with electrocoagulation of the pedicle was done under general anesthesia. A curvilinear longitudinal anterolateral aspect knee incision was given. On surgical exposure, there was a glistening dirty white firm tissue out-pouching along the lateral joint line communicating with peroneal entrapment (Figure 5).

**Figure 6:**
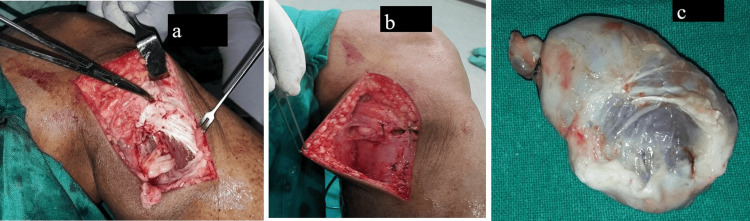
The pre-operative clinical image of the right knee shows (a) the cyst bed after excision with electrocoagulation of the cyst peduncle, (b) a loose approximation of the deep fascia, and (c) the excised cyst

The perineural cystic swelling was excised along with nerve decompression; the cyst pedicle was ligated, and electrocoagulation was performed at the base of the cyst (Figure 6).

A postoperative knee compression bandage with a foot drop splint was given. The patient was mobilized from the next day of the surgery with a knee brace support. 

Microscopic histopathologic examination of the cyst material revealed few macrophages with mucoid degeneration of ground substance consistent with a ganglion. There was no evidence of malignancy or mitotic activity. At six months post-operative follow-up, the patient was asymptomatic, maintaining full range of motion of the knee with normal stability of knee with improved sensations and power of foot. At one year, there were no signs of cyst recurrence. Dorsiflexion improved partially, likely due to the delayed treatment, although it enabled a bipedal gait with the support of a foot drop splint. The functional outcome was graded as good based on the Lysholm Knee Scoring Scale. A repeat NCV could have identified the progression of neural recovery, and a repeat ultrasound of the knee could have further confirmed if there was any cyst recurrence. However, as there was considerable symptomatic improvement, the consent for the suggested investigations was not given by the patient. Presently, we lack a longer follow-up period.

## Discussion

The most common cyst around the knee joint has been identified as Baker’s cyst, which accounts for almost one-third of all knee cysts. The synovial gangliomatous cysts are another common entity of benign asymptomatic nature. The synovial cysts are fluid-filled cysts with synovial lining [5]. The synovial gangliomatous cysts are normally filled with mucinous fluid within a fibrous capsule [3]. They may vary in size and are sometimes multi-loculated. Many times, the synovial and gangliomatous cysts are difficult to differentiate due to near connectivity to the joint [6]. The lower limb synovial gangliomatous cysts causing common peroneal neuropathy are uncommon and sparsely reported [3,7]. An intraneural cyst has been reported more commonly than an extraneural cyst [2,3]. The intraneural cyst normally has trauma to the articular branch of the nerve as an inciting factor [2,4]. The extraneural cysts are rare, and sporadic case reports are available in the literature [7]. The usual site of occurrence is along the proximal tibiofibular joint or the neck of the fibula [8]. The unlikely site of occurrence of extraneural peroneal compression neuropathy at the level of the supracondylar femur or proximal site of the popliteal fossa has also been reported [2]. An acutely presenting, non-traumatic, extra-articular peroneal cyst with foot drop in a 64-year-old male has been reported with near complete recovery [3]. We report a large, symptomatic, lateral soft tissue mass around the right knee in a 67-year-old male with no associated symptoms of instability or joint effusion. In our case, there was a “neglected” cyst with a foot drop of chronic duration of more than two months, which presented due to a non-traumatic cause. There was partial recovery with the need for a foot drop splint persisting.

The ganglion cyst usually presents with swelling and occasional pain in the knee. They may present with early sensory paresthesia to begin with due to nerve compression and may progress to motor weakness [4]. Patients with large cysts with persistent pressure may subsequently develop foot drop in the due course of time. The large swelling becomes more prominent on knee flexion.

Many theories have been proposed concerning the etiology of the cyst. The exact etiology is poorly understood [4]. Trauma to the knee has been postulated as one of the main predisposing factors. The most viable theory suggests a combination of knee degenerative changes with trauma. The motion of the knee acts as a pump to drive the synovial fluid into the cyst. The extra-articular cysts are hypothesized to originate from articulating proximal tibia-fibular joint [3]. A large, extra-articular, synovial gangliomatous cyst is an uncommon clinical finding and has been rarely reported. 

The radiograph of the affected joint assesses the joint congruity and associated arthritic changes contributing to the knee pain. The diagnosis of synovial cysts can be best supported by MRI, which gives information regarding the size and site of the cyst, its tract, and nerve pathology [1,2,5]. An ultrasound of the knee can also delineate the synovial cyst with high sensitivity and specificity with the advantage of it being an outpatient, portable, low-cost test, which can also detect dynamic changes in the swelling with movements of the knee. MRI can guide the surgical planning of treatment.

MRI shows a low intensity on T1-weighted images and a high intensity on T2-weighted images and can clearly show the communication of the swelling with the joint. Not all cysts produce a purely homogeneous signal on MRI due to hemorrhage or high protein content fluid, and not all cysts are associated with joint affection. Muscle denervation changes can also be easily identified on MRI in cysts involving the nerve sheath [5]. In case of swelling with mixed signals on MRI, a tissue diagnosis is imperative. MRI will be helpful to study and confirm the cyst, its tract, and its communication with the joint. 

The etiology of foot drop should be distinguished from an L5 peripheral nerve root lesion, which can be progressive and normally requires surgical management of the spine [2]. The preceding symptoms of sensory disturbances before the onset of foot drop are an important indicator for consideration of surgical management to prevent delayed neural deficit [3]. An intraneural peroneal cyst in a 58-year-old female was excised after about one month of foot drop presentation following the failure of the trial conservative measure. The paresthesia in the foot persisted even after a year of the surgery [8]. Normally, the peroneal cyst presents with marked weakness of ankle dorsiflexion movements. It disables the normal gait pattern leading to an early detection, and an immediate consultation is sought for the management in this acute phase of presentation. However, in our case, the presentation was late even though there was a persistent weakness, which had disabled the gait pattern. 

The conservative management of cysts with nerve compression along with individualized rehabilitation protocol has given marginal benefits with poor recovery outcomes [7]. The varied treatment suggested for cysts includes needle aspiration of the cyst, cyst excision, ligation of the cyst pedicle, and nerve decompression. The cyst extending into the soft tissues can be percutaneous, decompressed, and injected with steroids though chances of nerve damage and recurrences are high. A total cyst excision followed by electrocoagulation of the cyst pedicle has given consistent results with a very low rate of recurrence [3]. The risk of permanent nerve damage, infection, and joint stiffness are a few inherent complications, which need to be weighed upon for an individual plan of management [4]. The cyst excision through limited lateral exposure has also been recommended, along with nerve decompression and cystectomy. An endoscopic resection of the cyst has also been described. Endoscopic resection requires a clear understanding of anatomy, is highly technical, and is associated with risks of damage to the nerve, collateral ligaments, and the popliteus tendon [9]. The success of all procedures largely depends on adequate cyst management. The recurrence rate has been the lowest with an open approach.

In view of palpable large-size swelling with concomitant nerve entrapment, a cystectomy with nerve exploration, decompression, and electrocoagulation of the cyst pedicle was considered the better option to obviate the compressive aspects of cyst preserving the function of the joint.

## Conclusions

The lateral, extra-articular, perineural synovial cyst can be approached with open cystectomy and nerve decompression, with a guarded prognosis in a neglected swelling of long-standing duration. The delay in treatment may not allow the desired neural recovery following nerve decompression. An early intervention favors a better recovery outcome for both knee function and neuropathy. 
